# The Etiopathogenic Mosaic of Suicidal Behaviour

**DOI:** 10.3390/bs15010087

**Published:** 2025-01-18

**Authors:** Doinița Temelie-Olinici, Anton Knieling, Dan Vâță, Laura Gheucă-Solovăstru, Monica Neamțu, Mădălina Mocanu, Adriana-Ionela Pătrașcu, Vasile-Bogdan Grecu, Daniela-Anicuța Leca

**Affiliations:** 1 Department of Morpho-Functional Sciences II, “Grigore T. Popa” University of Medicine and Pharmacy, 700115 Iasi, Romania; doinita.p.olinici@umfiasi.ro (D.T.-O.); monica.neamtu@umfiasi.ro (M.N.); grecu_bogdan@ymail.com (V.-B.G.); 2 Forensic Science Department, Faculty of Medicine, “Grigore T. Popa” University of Medicine and Pharmacy, 700115 Iasi, Romania; 3 Department of Dermatology, “Grigore T. Popa” University of Medicine and Pharmacy, 700115 Iasi, Romania; dan.vata@umfiasi.ro (D.V.); solovastru.gheuca@umfiasi.ro (L.G.-S.); madalina.mocanu@umfiasi.ro (M.M.); 4 “Sf. Spiridon” County Clinical Emergency Hospital Iasi, 700111 Iasi, Romania; patrascuai@yahoo.com; 5 Department of Infectious Diseases (Internal Medicine II), Faculty of Medicine, “Grigore T. Popa” University of Medicine and Pharmacy, 700115 Iasi, Romania; daniela.leca@umfiasi.ro

**Keywords:** suicide, suicidal ideation, self-harm, etiopathogenic factors

## Abstract

Suicidality is among the most controversial concepts in multi-disciplinary studies worldwide, regardless of the form and approach. The etiopathological variability in suicidal ideation correlates with the heterogeneity of the clinical and behavioural patterns of self-harm attempts, which significantly impact the prognosis and quality of life of patients. The main objective of the present study was to identify and outline the spectrum of factors predisposing to suicide, with the whole suite of consequences and manifestations in ideation and behaviour. In this regard, the research literature of the last decade contains numerous articles dealing with the theoretical premises pertaining to both the statistical and the profoundly psychological and philosophical dimensions of suicide. The micro-environment favouring the clinical evolution of self-harm/self-destructive thoughts and attempts to the terminal, final act integrates individual medical-biological and psychological factors into the overall social reality. Knowledge of the whole etiopathogenic amalgam with clinical-evolutionary implications allows for the development of methods and tools for the early assessment and prevention of suicidal risk. At the same time, the present study aims to qualitatively focus on the subjective motivation declared by patients regarding the internal, individual catalyst of suicidal ideation and attempts on a predominantly psycho-social coordination.

## 1. Introduction

Since ancient times, suicidal ideation has been one of the most controversial concepts in multidisciplinary studies worldwide, regardless of the form and approach (Bachmann, 2018; Health Quality Ontario, 2017). Over the years, this topic has elicited a range of heterogeneous reactions, between two extremes: that of reprobation, and that of full acceptance and glorification (Woo et al., 2018; Michel et al., 2017). Over the course of life, suicidal thoughts, both passive and active, have a lifetime prevalence of about 9% (Bachmann, 2018; Health Quality Ontario, 2017). They are not actual behaviours (Chu et al., 2017). There are suicide thoughts that can never be communicated, directly or indirectly, regardless of their repetitiveness and intensity (Woo et al., 2018; Carrasco-Barrios et al., 2020). Therefore, there is support for the theory that suicidal ideation is one of the important predisposing factors for suicide (McHugh et al., 2019; Kang et al., 2020). In general, the conceptualization of motivation indicates the presence of a process that allows for organisms to self-regulate their internal and external environment by controlling stimuli (Chu et al., 2017; Conti et al., 2017). In the case of suicide, neither its motivational sources (Health Quality Ontario, 2017) nor its characteristic particularities are identified and understood (Woo et al., 2018; Kang et al., 2020). Some classical theories suggest the intervention of the directional and activational aspects of motivation, which are essential in removing significant stimuli and overcoming obstacles and constraints (Conti et al., 2017; Michel et al., 2017). In this context, the term self-harm encompasses both suicidal and non-suicidal attempts such as self-flagellation by cutting as a means to manage or cope with difficult emotional states (Health Quality Ontario, 2017; Woo et al., 2018; Kang et al., 2020; Subramanian et al., 2020).

In search of answers, the World Health Organization (WHO) defines suicide as “the act by which an individual seeks to physically destroy himself or herself with the more or less genuine intention of losing his or her life, while being more or less aware of his or her motives” (Subramanian et al., 2020; Carrasco-Barrios et al., 2020). In contrast, Schneidman considers this to be “the conscious act of self-annihilation, best understood as a helpless individual’s state of malaise, a state generated by a situation for which the suicidal act seems the best solution” (Bachmann, 2018; Pitman et al., 2017; Jakobsen et al., 2017; Baek et al., 2018).

In recent centuries, starting from Maris’ theory from 1981 according to which “suicide cannot be prevented until it is correctly conceptualized”, numerous etiopathogenetic and clinico-evolutionary patterns have been imagined and developed with the aim of improving the quality of patients at suicidal risk (Bachmann, 2018; Health Quality Ontario, 2017).

According to the “Diagnostic and Statistical Manual of Mental Disorders” (DSM-5), the diagnosis of “non-suicidal self-harm” can be distinguished from “suicidal behaviour disorder” or “suicide” (Carrasco-Barrios et al., 2020). However, looking in more detail at the terminology related to this pathology, there is a tendency to apply and respect the use of neutral notions such as suicide or completed suicide (Chu et al., 2017; Subramanian et al., 2020). In addition, one also opts for the inclusion of clues regarding the intentions, motivations, and outcomes of suicide Chu et al., 2017). These may include the following: attention, caring, protection seeking, comfort, or self-punitive purpose (Nigg, 2016; Jakobsen et al., 2017).

Every year, around one million people worldwide use some type of self-harm mechanism (Woo et al., 2018; Kang et al., 2020; Subramanian et al., 2020). According to the World Health Organization (WHO) report in 2015, the global annual suicide death rate is estimated to be about 10.7 per 100,000 individuals, which is 1.4% of all premature deaths. With regard to Romania, in contrast to the 1990s, when a suicide mortality rate of 9.47 per 100,000 inhabitants was identified, and in 2017 it slightly decreased to 9.16 (Chu et al., 2017; Carrasco-Barrios et al., 2020). While some epidemiological data report suicide as the third leading cause of death in the 10–24 age group, records from the US in 2014 ranked it second among the 12–34 age group, with over 11,000 suicides in 2012 (Nigg, 2016; Kang et al., 2020).

There are variations depending on age and background (Conti et al., 2017). Therefore, it is reported that 78% of all suicides occur in low- and/or middle-income countries, which correlates directly with socio-economic status and access to specialized medical assistance (Woo et al., 2018; Michel et al., 2017; Kang et al., 2020). It is also stated that other factors, such as demographic, educational, and/or biological factors, play a role in the dynamics of this parameter (Nigg, 2016). This also explains the diversity of self-harm methods practised, involving the use of hanging, self-poisoning, and shooting with different firearms (Subramanian et al., 2020; Pitman et al., 2017; Jakobsen et al., 2017; Baek et al., 2018).

Suicide is hypothesized to be associated with mental disorders manifested by negative hyperthymia, psychosis, and/or substance use (Chu et al., 2017; Espinet et al., 2019). Less relevant is the correlation with anxiety, food, traumatic history, or organic personality and/or mental disorders (Conti et al., 2017; Orsolini et al., 2020). With regard to endogenous personality disorders, significant correlations with suicidal risk have been demonstrated, especially for cluster B psychopathies (mainly borderline/emotional instability personality disorder) (Nigg, 2016; Bachmann, 2018). This interrelationship is also implicitly based on the high risk of developing depressive episodes that these cases imply (Bandelow et al., 2017; Kessler et al., 2020).

Suicide and suicidal ideation are markers of emotional distress, often associated with other significant psychological difficulties that have a major negative impact on clinical and therapeutic outcomes (Bachmann, 2018; Health Quality Ontario, 2017; Woo et al., 2018; Michel et al., 2017). According to the American Psychiatric Association (APA) Clinical Practice Guidelines, assessment of suicidal risk is a complex multi-axial process that involves corroborating all information about the patient’s ideation and behaviour, as well as psychosocial and family factors (Nigg, 2016; Michel et al., 2017; Jakobsen et al., 2017; Chu et al., 2017; Baek et al., 2018).

## 2. The Main Etiopathogenic Mechanisms of Suicidal Behaviour

**Figure 1:**
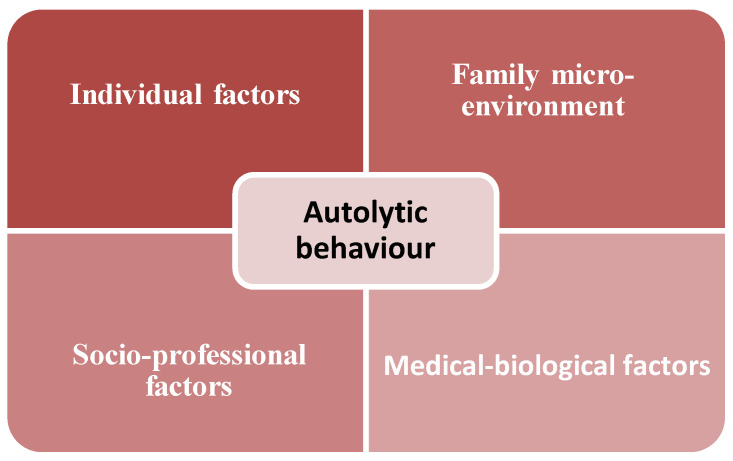
The main factors defining self-harm behaviour.

Suicide, as an object of multidisciplinary scientific study, presents both sociological and anthropological, as well as psychological and psychopathological, valences (Bachmann, 2018; Woo et al., 2018), with each of these heuristic domains providing important insights into self-harm behaviour (Figure 1) (Nigg, 2016; Chu et al., 2017; Kessler et al., 2020).

The multifactorial heterogeneity of suicidal behaviour brings to the fore a mix of predisposing and contributing risk factors (Nigg, 2016; Chu et al., 2017). These include climatic conditions (Lunde et al., 2018), demographic factors, local–traditional and socio-occupational factors, family history and behaviour, personal history of suicide attempts, the existence of serious prognostic social conditions, and reactive states after major stressors (Schaffer et al., 2015; Conti et al., 2017; Kessler et al., 2020). In the absence of a support network, their action is synergistic in the manifestation of self-injurious ideation (Bandelow et al., 2017; McHugh et al., 2019).

### 2.1. Climatic Conditions

Climate change is an increasing risk factor for ecosystems and human health (Benard et al., 2015; Rumble et al., 2018). To some extent, these changes have been correlated with the seasonal distribution and propensity of depressive episodes, especially in bipolar disorder, but also in cases of other hyperthymic negative syndromes (Benard et al., 2015; Nigg, 2016; Baek et al., 2018). In this context, the frequent association of suicide and/or suicide attempts with certain weather conditions is identified (Schaffer et al., 2015; Bachmann, 2018; Lunde et al., 2018). Statistical data aim to correlate suicidal ideation with either acute day-long or chronic month-long or longer changes in climatic factors (Benard et al., 2015; Chu et al., 2017; Kessler et al., 2020).

Over the years, there is an interdependence (Bachmann, 2018) between autolysis and reduction in atmospheric pressure, seasonal asymmetry—with exacerbations in the spring months, especially April (Lunde et al., 2018), and with reductions in the autumn months (Rumble et al., 2018)—temperature variation, circadian rhythm—with a peak in the evening in the young population and another in the morning for the elderly population (Benard et al., 2015; Health Quality Ontario, 2017; Woo et al., 2018)—atmospheric pollutants, and the presence or absence of sun (Chu et al., 2017; Bandelow et al., 2017; Kessler et al., 2020).

All these factors influence the synthesis and action of melatonin, serotonin, and cor-tizol, with serious repercussions on sleep quality and quantity (Benard et al., 2015; Jakobsen et al., 2017; Littlewood et al., 2017). In other words, insomnia, nightmares, and sleep deprivation are common risk factors for suicide, irrespective of the presence or absence of symptoms suggestive of depression (Nugent et al., 2011; Littlewood et al., 2017). It is suggested that in these situations the use of chronotherapy has beneficial effects in significantly reducing the risk of suicide (Jakobsen et al., 2017; Rumble et al., 2018).

Awareness of the involvement of these factors in the characterization of suicidal behaviour is an important step in the algorithm of stratification of self-harming behaviour in the general population (Bachmann, 2018), with considerable improvement in both preventive and therapeutic methods (Kessler et al., 2020).

### 2.2. Demographic Factors

At the population level, ethnic variations may be significantly correlated with genetic differences in suicide susceptibility, explaining different epidemiological values and thus indirectly confirming the neurobiological background (Nugent et al., 2011; Cheref et al., 2015; Chu et al., 2017; Peng et al., 2024). At the population level, the initiation and development of prevention and intervention strategies have revealed that some ethnic variations may be significantly correlated with genetic differences in suicide susceptibility (Cheref et al., 2015; Forte et al., 2018; Peng et al., 2024). Obtaining different epidemiologic values may indirectly confirm the neurobiological background (Nigg, 2016; Baek et al., 2018). On the other hand, disparities perpetuated by structural racism may lead to disparities in mental health equity in diverse minority communities (Cheref et al., 2015; Bandelow et al., 2017; Forte et al., 2018; Peng et al., 2024). 

In recent years, the prevalence of extreme self-destructive suicidal behaviours has increased significantly among children and adolescents (Cheref et al., 2015; Stewart et al., 2019). In this respect, suicide is reported as the second most common cause of death in the 12–15 age group (Sitnik-Warchulska & Izydorczyk, 2018). Approximately 8% of American high school students and 30% of Polish teenagers report suicidal behaviour (Angelakis et al., 2019). In 2016, a study conducted by Inchly et al. reports that 4–52% of 15 year olds exhibit violent behaviour against others (Health Quality Ontario, 2017). A development of destructive behaviour is noted, especially among the female population (Conti et al., 2017; Forte et al., 2018; Peng et al., 2024).

Most often, suicidal ideation identified in young people is correlated with extreme aggression (Nugent et al., 2011; Health Quality Ontario, 2017; Forte et al., 2018), such as interpersonal violence, considered the third most common cause of death in the 10–29 age group (Conti et al., 2017; Rappaport et al., 2017; Peng et al., 2024).

It is observed that the prevalence of suicide rates increases distinctly in adolescence and early adulthood (Nugent et al., 2011; Wang et al., 2017; Forte et al., 2018). In contrast to males who commit suicide using highly aggressive methods, females resort to minimally aggressive means by resorting to suicide threats and attempts (Peng et al., 2024).

### 2.3. Socio-Professional Factors

Researchers in the field of narrative theory support contemporary studies that emphasize the importance of using individual perception and its relationship to the manifested behaviour (Conti et al., 2017; Rappaport et al., 2017; Woo et al., 2018). However, the role played by biological and socio-cultural factors should not be underestimated (Littlewood et al., 2017; Underwood et al., 2018; Acuña Caicedo et al., 2020; Carrasco-Barrios et al., 2020, p. 23).

At the end of the 19th century, in an attempt to establish certain statistical patterns (Schaffer et al., 2015), the first major contribution to the socio-cultural component of suicide was made by Durkheim (Table 1), who divided suicide socially into three categories: egoistic, altruistic, and anomic (Espinet et al., 2019).

The first psychological insight into suicidal motivation belongs to Sigmund Freud, who equated the suicidal act with an aggression directed towards an intraprojected, ambivalent, loved “object”. At the same time, he argues that there is no suicide without a prior desire to take the life of another (Michel et al., 2017). People who make suicidal choices exhibit hostility and tension, similar to that observed among people who use violence against others (Benard et al., 2015).

The interpersonal theory of suicidal behaviour demonstrates the increased susceptibility to experiencing “suicidal desire” among individuals with low social support, low belongingness, and individuals who develop a strong sense of being a burden to those around them (Nigg, 2016; Chu et al., 2017; Baek et al., 2018; Subramanian et al., 2020). In addition, this theory shows that active action on suicidal thoughts, with ultimate outcome, is conditioned by a third factor, namely the acquisition of a sense of fearlessness in the face of death, with increased pain tolerance (Wang et al., 2017; Kessler et al., 2020).

In the pilot socio-cognitive outpatient study developed by Duarté-Vélez et al. in the adolescent Latino population, reliable improvements in prognosis and quality of life of patients at risk of suicide are obtained (Stewart et al., 2019; Forte et al., 2021).

Given that among adolescents, risk factors are similar to those described for extreme self-destructive and aggressive behaviour (Wang et al., 2017), the idea of a generalized destructive syndrome is supported (Chung et al., 2017; Pitman et al., 2017; Woo et al., 2018). In addition, the repetitive and ongoing nature of suicidal and violent behaviours indicates the need to treat them as separate nosological units (Schaffer et al., 2015; Macleod et al., 2018; Fritz et al., 2018; Forte et al., 2021).

Sitnik-Warchulska and Izydorczyk suggest that all violent behaviour has a so-called self-destructive dimension (Sitnik-Warchulska & Izydorczyk, 2018; John et al., 2018). Consequently, they are associated with social sanctions that include personal costs such as isolation and loss of freedom (Fritz et al., 2018).

In the last decade, important links are reported between cyber-bullying and suicide (Angelakis et al., 2019; Bernert et al., 2020). Internet use has a mixed effect on the well-being of children and young people (Woo et al., 2018; Forte et al., 2021). The identification of a large number of suicides facilitated by social media calls for increased efforts to investigate the causal relationship between internet use and self-harm, suicide (Sequeira et al., 2019; Liu et al., 2020; Forte et al., 2021).

There remains much controversy about the role of the internet in young people’s self-harm (Forte et al., 2021). There is an assumption that accessing certain forums poses an increased risk. Internet addiction and pro-suicide websites are major risk factors in facilitating suicidal behaviour (Michel et al., 2017; Kang et al., 2020). Recent studies on the interrelationship between online content and suicide identify changes over time in searches for self-harm-specific content, with a predominance of graphic images (Subramanian et al., 2020; Pitman et al., 2017). Half of these sites contain videos providing information on self-harm methods with negative influences on self-harm behaviour. However, analysis of many websites also identifies positive aspects, such as advice on seeking first aid (Sequeira et al., 2019).

Excessive internet use and internet addiction have a largely negative impact on adolescent development (Forte et al., 2021). Suicidal ideation is associated with more than 2–5 h/day of internet use (Liu et al., 2020). Some negative aspects of internet use can be mitigated by introducing psycho-educational prevention programmes in schools and implementing social-media platforms that create counselling and prevention resources (Macleod et al., 2018; Forte et al., 2021).

It can be said that the positive or negative influences of the online environment are perceived differently, depending on the culture of the patient and the mental health professional (Dickson et al., 2019; Liu et al., 2020). The results of this research also depend on the design and quality of individual studies. In this regard, systematic reviews by many researchers point to the need for more rigorous methodologies, focusing on mediating and moderating factors in optimizing the potential benefits of the internet among young people (Macleod et al., 2018; Sarchiapone et al., 2018; Sequeira et al., 2019).

### 2.4. Local–Traditional Factors

As defined by the APA (American Psychological Association) in 2003, culture is “one of the belief and value orientations systems that influence social customs, norms, practises and institutions, including psychological processes (language, care practises, media, educational systems) and organizations (media, educational systems)” (Liu et al., 2020; Corke et al., 2021). Although it is embedded in everyday life, it frequently seems invisible to those who study and treat human behaviour (Chung et al., 2017; John et al., 2018; Dickson et al., 2019). Multicultural approaches explain the differences in views of minorities, but also those related to gender, sexual orientation, socio-economic status, and disability (Fritz et al., 2018; Sequeira et al., 2019).

The increasing diversity of populations in the United States and the proximity of countries due to globalization requires cultural competence in providing psychological interventions (Woo et al., 2018; Michel et al., 2017; Kang et al., 2020; Subramanian et al., 2020; Pitman et al., 2017). The majority of therapeutic requests are made on specific ethnic or cultural groups, which may not apply globally (John et al., 2018; Dickson et al., 2019; Sequeira et al., 2019).

Culturally specific treatments, i.e., those therapies that “*incorporate modifications to psychotherapy processes and/or content with the intention of increasing congruence between the patient’s ethnocultural worldview and evidence-based practice*”, are found to be more clinically beneficial than those that ignore cultural norms (Corke et al., 2021). These observations are also supported by meta-analyses identified in the research literature (Pitman et al., 2017; Dickson et al., 2019; Yates et al., 2019).

In the case of adolescents, several emerging studies addressing population-specific cultural gaps identify high levels of suicidal ideation among African Americans (Forte et al., 2018; Kessler et al., 2020). The implementation of pilot trials in the prediction and reduction of self-harm behaviour demonstrates that a diverse sample does not necessarily indicate the use of culturally tailored therapeutic principles (Angelakis et al., 2019; Carrasco-Barrios et al., 2020).

Reductions in depressive symptoms and post-treatment suicidal ideation are also identified in protocols adapted to the adolescent culture of Puerto Rico and protocols based on ecological validity criteria (Sarchiapone et al., 2018; Yates et al., 2019). Moreover, in the case of gay adolescents, the same protocols are effective in integrating conflicting core beliefs about spirituality, sexuality, and family values/principles (Ringer et al., 2018; Rumble et al., 2018; Angelakis et al., 2019).

Some culturally focused and tailored therapeutic management schemes mainly target major risk factors for suicide such as depression (Orsolini et al., 2020), behavioural problems, post-traumatic stress symptoms, and family functioning, without focusing on suicidal thoughts and behaviours (Dickson et al., 2019; Corke et al., 2021). Thus, a better insight into the belief systems of the family and the patient can be gained (Ringer et al., 2018; Rumble et al., 2018).

When working with patients from minority populations, it is necessary to use an eco-logical approach in understanding personal, family, socio-environmental, and cultural risk and protective factors (John et al., 2018; Sequeira et al., 2019). Environmental stressors, such as immigration background, violence, discrimination, socio-economic difficulties, and language barriers, can be highlighted and understood as predictors that facilitate case conceptualisation with the alleviation of feelings of guilt (Chung et al., 2017; Ringer et al., 2018; Arensman et al., 2019). It is very important to validate stressful experiences and family strengths that would help them “survive” situations prior to a crisis or suicide attempt (Fritz et al., 2018). Such an approach is also important in increasing adherence to treatment in under-age populations, often reluctant to seek mental health services (Schaffer et al., 2015; Macleod et al., 2018; Rumble et al., 2018).

Although therapeutic alternatives for minority patients with suicidal thoughts and behaviours are limited, the prospect of a multicultural, evidence-based approach shows promising results and benefits (Wang et al., 2017; Dickson et al., 2019; Forte et al., 2021). Combining individual characteristics—culture and therapeutic preferences—and clinician’s expertise may provide the best option for increasing adherence and treatment efficacy for minority youth (Benard et al., 2015; Espinet et al., 2019; Carrasco-Barrios et al., 2020).

### 2.5. Medical-Biological Factors

Identifying suicidal risk remains one of the most difficult diagnostic challenges in psychiatry (Wang et al., 2017; Woo et al., 2018), often targeting patients who exhibit markedly disruptive behaviour (Conti et al., 2017; Corke et al., 2021).

According to population-based studies, reporting in the general population reveals that suicide is 3–12 times more common in psychiatric patients (Ringer et al., 2018; Arensman et al., 2019). In this regard, among the main disorders associated with autistic behaviour are affective disorders—major depression and bipolar disorder, schizophrenia, and alcohol abuse and dependence—personality disorders—borderline and antisocial, organic disorders—epilepsy and dementia, and anxiety disorders—post-traumatic stress disorder, unipolar depression (MacArthur et al., 2018; Stewart et al., 2019; Orsolini et al., 2020).

Among the most important predictors of suicide are history of previous attempts and depression (Ringer et al., 2018). Thus, studies of the Chinese population estimate prevalence values of 0.4% for patients with bipolar disorder and 1.4% for those with major unipolar depression (Orsolini et al., 2020).

Attempted self-harm in the medical history is the risk factor with the greatest impact on prognosis and the only one with real predictive value, as it is known that one in two attempts is actually a relapse (Schaffer et al., 2015; Ringer et al., 2018). Depending on their frequency, the occurrence of new attempts or even suicide can be predicted (MacArthur et al., 2018; Spillane et al., 2018; Amin et al., 2021).

At the opposite end, the existence of a sense of social adequacy, pregnancy, children in care, strong religious beliefs, and the presence of social support are protective factors against self-harming behaviour (Chung et al., 2017; Lim et al., 2019).

According to Rockville, in 2014, the National Institute of Mental Health and the Research Prioritization Task Force’s aspirational Goal #6 states the following: “*Ensure that people who have attempted suicide can receive effective interventions to prevent further atempts*” (Spillane et al., 2018; Hill et al., 2020).

It is estimated that about 45–64% of suicide victims have a pathology associated with depression (Sarchiapone et al., 2018). Conversely, 15% of people with depression end up committing suicide (Spillane et al., 2018). Depressive states occur in many mental illnesses and may be accompanied by self-harming behaviour (Kessler et al., 2020), which is why depression must be treated as a genuine medical-psychiatric emergency (Rumble et al., 2018; Sarchiapone et al., 2018; Probert-Lindström et al., 2020).

Social problem-solving and depression can interact to significantly influence suicidal thinking and behaviour (Spillane et al., 2018; Stewart et al., 2019). Thus, poor coping is directly correlated with depression, suicidal ideation, and suicide attempts (Lunde et al., 2018; Amin et al., 2021). Multiple epidemological research suggests that, unlike the general population, depressed people have a much lower ability to deal with the various problems they face (Rumble et al., 2018). Moreover, resolving them can have a significant impact on depressive symptoms (Lim et al., 2019). The amount of relevant and irrelevant solutions of daily problems is directly associated with depressive symptoms, lack of perspective, and suicidal behaviour (Stewart et al., 2019; Hill et al., 2020).

Previous research suggests that those who develop suicidal behaviour are incapable of thinking and finding alternative solutions to their problems and suffering (Schaffer et al., 2015; Jakobsen et al., 2017; Conti et al., 2017). Among vulnerable populations, understanding how problem-solving interacts with depressive symptoms can influence suicidal behaviour (Schaffer et al., 2015). In this way, potential checkpoints for specific therapies can be identified (Lim et al., 2019).

The reduction in depressive symptoms correlates with a reduction in suicidal ideation (McHugh et al., 2019), a result also confirmed by some research that supports the use of MEPS (Means Ends Problem Solving) in interpersonal conflict resolution (Hill et al., 2020).

A psychological autopsy study conducted on a cohort of 571 suicides found that only about 22% of the cases presented to health professionals involved suicide (Arensman et al., 2019; Probert-Lindström et al., 2020). Similarly, in the USA and Australia, only one-fifth of the 80% of patients who seek the help of professionals report attempted suicide (Ma et al., 2019; Zhang et al., 2019).

When chronic life-threatening cancer or cardiovascular diseases occur in the family, the stress they cause can reinforce family dysfunction (Arensman et al., 2019). As a result, family members display helplessness and hopelessness synergistically with the tendency to avoid expressing conflict, hostile feelings, or overprotectiveness of the family member suffering from a particular disease (MacArthur et al., 2018). This may cause the family to limit their interactions and social relationships as much as possible and promote the development of vegetative and psychosomatic symptoms secondary to hidden emotions (Zhang et al., 2019). From a systemic perspective, the illness becomes a fixed strategy and an element of family bonding (Ma et al., 2019). Such cross-generational patterns of relationships and projections can be identified especially in young women with suicidal ideation (Lunde et al., 2018; Mars et al., 2019). They manifest excessive involvement in family care (Madsen et al., 2017).

The magnitude and direction of the relationship between obesity and suicidal ideation/suicidal behaviour is unclear (Lim et al., 2019; Mars et al., 2019). In this regard, several large epidemiological studies report an indirect causal relationship between body mass index (BMI) and completed suicide, such that individuals with a high degree of obesity have a lower risk of death by suicide compared to others (Ma et al., 2019; Hill et al., 2020). In contrast, some research reveals direct interdependence between these two factors: body mass index value and frequency of suicide attempts (Madsen et al., 2017; Zhang et al., 2019; Kim et al., 2020).

Given that conventional risk factors such as alcohol consumption, social and marital status, and firearm ownership do not fully explain the association with the negative prognosis of suicide, other variables that may influence suicidal ideation and behaviour in the overweight population are examined (Mars et al., 2019). Thus, analyzing the context of the painful and challenging experiences of these patients, a correlation between undergoing bariatric surgery and increased suicidality can be observed (Madsen et al., 2017).

Examining the quadratic relationship between body mass index and suicidal ideation is an important step, given the increased consequences on psychosocial health, including depressive symptoms observed especially in extreme cases of obesity (Zhang et al., 2019).

Two directions for designing strategies aimed at solving different types of problems are identified: the vertical axis, determined by generational patterns, including biological inheritance, genetic marker, congenital disabilities, and temperamental and behavioural predisposition (Madsen et al., 2017); and the horizontal axis, characterized by emotional, physical, cognitive, and interpersonal changes in individuals over the life course in a specific social context (Arensman et al., 2019).

### 2.6. Family Micro-Environment

Over time, the diagnostic and therapeutic protocols specific to patients with ideations and/or self-limiting tendencies have been continuously adapted (Bachmann, 2018; Health Quality Ontario, 2017; Woo et al., 2018; Michel et al., 2017; Kang et al., 2020) with the inclusion of the family in the management of emotional reactions (Ringer et al., 2018; Ma et al., 2019; Subramanian et al., 2020).

Over the years, a number of hypotheses have been put forward and addressed that support the existence of the following: a *specific structure of the family system*—identifying numerous experiences of loss or neglect and/or changes in family structure (MacArthur et al., 2018); a *family constellation*—intra-family relationships experience multiple difficulties; *family projections*—associated with aggressive or suicidal strategies and/or behaviours; and a *lower level of overall family adjustment* (Mars et al., 2019; Amin et al., 2021).

Some research shows that the risk of suicide is increased in patients who have the family rank of middle sibling, associated with increased flexibility and ambivalence (MacArthur et al., 2018; Amin et al., 2021). Therefore, it is assumed, according to the results of McGoldrick et al., that the middle sibling position is associated with a certain degree of anxiety, fuelled by continuous comparisons with the other siblings, which predisposes to the occurrence of self-harm (Madsen et al., 2017; Lim et al., 2019). Similar results are obtained by Kirkcaldy, Richardson-Vejlgaard, and Siefen, researchers who believe that this relationship can be highly modulated by siblings (Ma et al., 2019; Hill et al., 2020).

Often, the family history of patients who develop violent behaviour seems similar to that of patients with suicidal ideation (Kim et al., 2020). Thus, it is found that the family history of aggressive young women is dominated by emotional problems induced by their fathers and/or brothers (Sun & Zhang, 2018). In addition, another characteristic of these families is the projection of either alcohol abuse or domestic violence (Ruderfer et al., 2020).

Experiences of physical violence, rejection, and assistance to violence are among the significant predictors of criminal behaviour (Lunde et al., 2018).

The observations made by both Howell et al. and by Fryers and Brugha indicate that these patterns are frequently associated with the use of violence among children and adolescents (Sun & Zhang, 2018; Kim et al., 2020), negatively affecting their ability to give and receive support from others (Madsen et al., 2017; Mars et al., 2019).

Ambivalence can promote the emergence and development of short-term coalitions with either parent (Angelakis et al., 2019). According to Esfandyari, Baharudin, and Nowzari, it can be argued that such “partnerships” can result from a constant tension in the parents’ relationship, a relationship that is observed and analyzed discordantly by the child (Macleod et al., 2018). In turn, the child intervenes in the parental conflict by carrying out the so-called triangulation process (Madsen et al., 2017).

This relational model, based on a low level of family adaptation, also characterizes the families of young people who display violent and self-destructive behaviour (Madsen et al., 2017). Thus, their family environments are dominated by open conflicts based on excessive alcohol consumption and violence. However, in the case of young people who develop only violent behaviours, close relationships are identified to a certain extent: “*the style of relationships is inadequate and ineffective, which could lead to ambivalence and a tendency to reinforce aggressive patterns as the only forms of relationships available*” (Huang et al., 2020).

It is often believed that the presence of suicide attempts in the family history correlates directly with the risk of suicide in subsequent generations (Sun & Zhang, 2018). Some observational studies also draw attention to the interdependence of violence/aggression–suicide (Mars et al., 2019). Thus, the existence of a case of suicide in one family is associated with the occurrence of aggressive behaviour in subsequent generations and vice versa. This tendency is supported and/or amplified by a number of biological and psychosocial factors (Bernaras et al., 2019).

In line with these findings, some clinical research points out that risk factors for self-harming behaviour in adolescents include both strained relationships or lack of inter-familial bonds and mental distress identified mainly in mothers (Fritz et al., 2018). The physical absence of fathers and emotional imbalances of mothers may prevent the expression of feelings of fear, which could direct anger towards the self (Bernaras et al., 2019).

Identifying and describing individual and family patterns and elucidating the meaning attributed to destructive behaviours are crucial in prevention and effective therapy (Fritz et al., 2018; Gallagher & Miller, 2018). In line with transgenerational family therapy, supported in particular by Bowen’s theory, it is essential to recognize, in these patterns, elements specific to the family environment such as the relationships between family members ((Bernaras et al., 2019; Ruderfer et al., 2020). The latter have a prognostic value for the suicidal and/or violent behaviours developed later, thus facilitating diagnostic and therapeutic management (Sun & Zhang, 2018; Huang et al., 2020).

This supports the idea that no individual can be understood and helped in an isolated way (Gallagher & Miller, 2018). It must be kept in mind that they are familial emotional units. Kerr and Bowen’s results suggest that clinical manifestations occur during intense and prolonged periods of family tension. In addition, the way in which the family approaches and manages different conflict situations is defining (McHugh et al., 2019). Thus, four patterns are postulated in relation to the problems identified in a family: marital conflict, good child focus, spousal dysfunction, and emotional distancing (Ludwig & Dwivedi, 2018). The evolution of each individual depends on their ability to separate their thoughts and feelings from those of others (Huang et al., 2020).

Numerous observations show that family relationship patterns are passed on to subsequent generations, primarily through the mechanism of emotional triangulation: two family members reduce tension by coalescing with a third member of the same family (Madsen et al., 2017; Ludwig & Dwivedi, 2018). Likewise, these interactions can be transferred from parent to child—as part of the family design process and/or repeated across generations—in a process of multigenerational transmission (Bernaras et al., 2019; Huang et al., 2020; Liu et al., 2020).

Contemporary research conducted by Olson confirms the importance of closeness and distancing identified in family interactions. Thus, his “Circumplex” model suggests that prognosis depends on family cohesion and flexibility (Levey et al., 2019).

Current narrative-based therapeutic concepts support transgenerational family theories (Sitnik-Warchulska & Izydorczyk, 2018; Levey et al., 2019). The latter address the use of genograms—pictorial diagrams of family data, including their structure and functional patterns, corresponding to the last three generations—in examining and identifying the role of family projections in the development of self-harming behaviour (Huang et al., 2020; Liu et al., 2020; van Bergen et al., 2021).

Clinical algorithms based on the analysis of these genograms can differentiate the behavioural characteristics of suicidal and violent youths (Bernaras et al., 2019). In this respect, the latter may have a challenging family history and a tendency to break rules and regulations, such as domestic violence, alcohol abuse, various serious illnesses, and/or difficulties in expressing emotions to other family members (Ludwig & Dwivedi, 2018; Ma et al., 2019). In contrast, aspects of this family pattern are much less frequently identified in young people with self-destructive tendencies (Levey et al., 2019; Yates et al., 2019).

In cases of girls with self-harming attempts and/or ideation, critical episodes are associated and directly correlated with certain family events or changes in the family environment such as loss of a loved one, divorce, reconstruction of the family system, diagnosis of cardiovascular and/or oncological diseases in many family generations, absence of a biological parent—frequently the father—and the emotional problems of the mother (Bernert et al., 2020; van Bergen et al., 2021).

A similar family pattern is present, albeit at a much lower intensity, in girls who use interpersonal aggression. Therefore, in order to re-establish a relative balance, the family micro-environment has to face new emotions that are difficult to manage and take different types of actions. So-called family resilience frequently faces difficulties in constructively adapting and using support to reinforce behaviours that may be maladaptive because of their durability and low susceptibility to change (Gallagher & Miller, 2018; Stanley et al., 2018).

The results of further research suggest that, in both groups of girls, a certain “readiness for destruction” is identified (van Bergen et al., 2021). Often only the use of one mechanism is indicated. However, no clear answers have yet been given to the question “what drives adolescent girls to extreme aggression or self-destruction?” (Stanley et al., 2018).

In both of the above family patterns, it is assumed that there would be a strong tendency for intra-family conflict to recur in the following generations (Levey et al., 2019). In other words, some family projections are thought to promote the creation of triangular relationships in which the child is in coalition with one parent against the other. This can lead to so-called triangulation, i.e., the development of overly strong relationships with the parent with whom the child is in coalition, resulting in a hidden conflict of loyalty and emotional ambivalence between what comes from the child him/herself and what comes from the identification of hostile emotions towards one of the parents (Bernert et al., 2020).

It is easy to understand why destructive behaviours of one family member have a significant impact on the family as a whole (van Bergen et al., 2021). They induce a reactive mosaicism of fear, guilt, anger, powerlessness, and social ostracism, feelings and beliefs strongly reinforced in the family schema. The ’family transaction’ pattern is passed on to subsequent generations (Stanley et al., 2018).

Also, these genograms, which follow Bowen’s family systems theory, are reliable psychosocial images of patients, regardless of their age and occupation (Angelakis et al., 2019). Nogueira et al. argue that they are genuine tools that can be used successfully in general practice, even in individual approaches characteristic to adolescents and children (Sitnik-Warchulska & Izydorczyk, 2018; Bernert et al., 2020). Thus, with their help, clinicians can reveal various contexts and events specific to family history, with significant implications both in prevention and in therapeutic effectiveness (Yates et al., 2019; van Bergen et al., 2021).

Although family transmission patterns of certain behaviours and inter-familial relationships are aspects that are within the realm of evidence regarding their relationship with potential suicidal manifestations (Sitnik-Warchulska & Izydorczyk, 2018), the literature is lacking in comprehensive information that takes into account all the variables that may be involved in this causal dynamic (Stanley et al., 2018; Angelakis et al., 2019). Thus, the need to correlate the bio-psycho-social dimensions of situational attitudinal patterns, in terms of highlighting the relationship between the depressive/dysthymic background, the accentuated personality traits, the addictive profile, and the relation to the family relational context emerges (Bernert et al., 2020; van Bergen et al., 2021).

A premise of this line of discussion is the clinical observation of numerous situations in which the suicidal motivation reported by patients with various types of depression is characterized by a series of factors prevailing in the family environment in all its multidimensionality (Ludwig & Dwivedi, 2018; van Bergen et al., 2021).

A fundamental problem is that the psychogenogram is a time-consuming and difficult assessment method to apply to a large target population, so that relevant results can be obtained also by correlating with other determinants of suicidal behaviour and to obtain meaningful correlations for the most rapid preventive approach (Bachmann, 2018; Health Quality Ontario, 2017; Woo et al., 2018). However, in the assessment and management of individualized cases, an in-depth exploration of the family relational background and patterns of interaction and behaviour, often passed down through generations, is extremely useful, as it can reveal predictors of suicidal behaviour or predisposition to depression and addictive, anxious, and dysfunctional behaviours that may precipitate self-harming actions over time, aspects that would not be revealed by a classic patient interview alone (Stanley et al., 2018; Levey et al., 2019).

## 3. Discussion

Suicidal behaviour is the result of a complex interaction between genetic vulnerability, endo- and exogenous stressors, underlying psychopathology, and socio-cultural aspects (Zhang et al., 2019). Therefore, it can be argued that the pathophysiological mechanisms are not yet fully understood, as they are incompletely investigated (Fritz et al., 2018). The establishment of clinical-evolutionary models of suicidal behaviour, starting from the correct and thorough identification and analysis of the favouring and predisposing risk factors, will guide the efforts to conceptualize all cases, with improvements in both prognosis and quality of life (Stanley et al., 2018; Levey et al., 2019).

**Figure 2:**
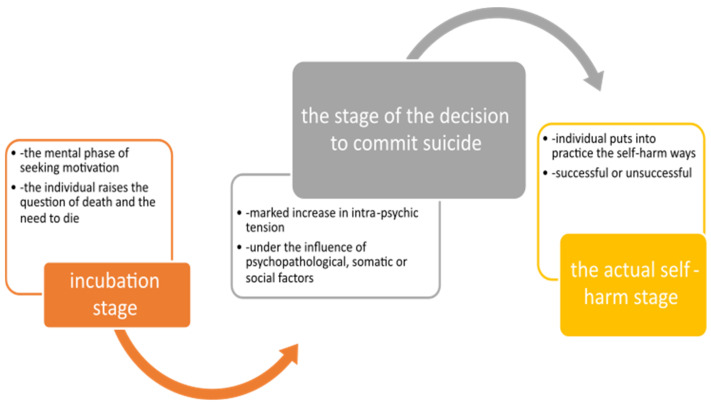
Evolution of the suicidal process.

Several aspects of suicidal behaviour can be distinguished (Figure 2) (Stanley et al., 2018). A first component is the suicidal ideation characterized by the different expression of thoughts without an autolytic purpose (Bachmann, 2018; McHugh et al., 2019). Another element is the threat of suicide which is constituted as the oral or written expression of the intention to commit suicide, without the presence of a clear-cut desire to commit this act (Bernert et al., 2020; Probert-Lindström et al., 2020).

Suicide attempts that delimit the notion of parasuicide are those self-injurious actions carried out either with the real intention to commit suicide, failed suicide (McHugh et al., 2019), or with the intention to transmit various states or messages to the entourage (Bachmann, 2018; Health Quality Ontario, 2017; Woo et al., 2018; Michel et al., 2017; Kang et al., 2020). The number and type of suicide attempts are important predictors of suicide (Nugent et al., 2011; Nigg, 2016).

Complete suicide represents the terminal and irreversible element of autolytic behaviour (Bachmann, 2018; Health Quality Ontario, 2017; Woo et al., 2018; Michel et al., 2017; Kang et al., 2020) in which various life-threatening injuries are produced with the clear prior intention to die (Benard et al., 2015; Sun & Zhang, 2018; Kim et al., 2020).

In the last 5 years, the statistical power of different types of suicide predictors has been examined by a series of high-quality meta-analyses (Conti et al., 2017; Michel et al., 2017; Rappaport et al., 2017; Bachmann, 2018; Woo et al., 2018). Both Franklin et al. and Ribeiro et al. conducted systematic reviews of longitudinal studies that reported the predictive power of a broad spectrum of risk factors for self-harm, including suicidal ideation and suicidal behaviour (Sitnik-Warchulska & Izydorczyk, 2018; Carrasco-Barrios et al., 2020). They concluded that even the most highly rated and reliable risk factors for suicide “provide only a marginal improvement in diagnostic accuracy over chance” (Underwood et al., 2018; Acuña Caicedo et al., 2020; Probert-Lindström et al., 2020).

Other authors have used meta-analyses to examine the predictive power of suicide risk validity scale, concluding that “the scales lack sufficient evidence to support their use”, “are not clinically useful”, and “do not meet the requirements for diagnostic accuracy” (Pitman et al., 2017; Jakobsen et al., 2017). More recently, Belsher et al. synthesized 17 suicide prediction models, developed by using both the training-exploratory and the testing-validation stages, and concluded that their accuracy is close to zero (Baek et al., 2018; Hill et al., 2020; Kang et al., 2020; Subramanian et al., 2020).

Evidence has been found that suicide prediction models perform better in general community/non-mental health settings and in actual hospitalization settings (Chu et al., 2017; Ma et al., 2019). Although these findings were incidental and may not be replicated, it is possible that suicide risk factors are more significant in settings where risk factors are less prevalent, such as a psychiatric diagnosis in the general community, and when more accurate and detailed risk profiles are available, such as in a hospital (Pitman et al., 2017; Rappaport et al., 2017; Zhang et al., 2019).

Suicide prediction models seem to perform less well after suicide or self-harm attempts (Pitman et al., 2017). The most obvious reason for this is that suicide or self-harm attempts, which were the only risk factor frequently included in the determination of autolytic behaviour, cannot be used in the suicide prediction model because both suicides and survivors have this risk factor (Macleod et al., 2018; Acuña Caicedo et al., 2020).

Over the years, the synergistic role of both genetic factors and exogenous stressors has been indicated: deficiencies in interpersonal and professional relationships, financial instability (Lunde et al., 2018; Spillane et al., 2018), and endogenous-psychiatric disorders, epigenetic factors, impaired stress response involving the hypothalamic–pituitary–adrenal (HPA) system, monoaminergic neurotransmitter systems, especially serotonergic neurotransmitters (Jakobsen et al., 2017; Ludwig & Dwivedi, 2018), and specific neurotrophins such as brain-derived neurotrophic factor (BDNF) (Rumble et al., 2018; Ruderfer et al., 2020).

There is no doubt that the etiopathogenesis of suicide and implicitly of self-harm is related both to the presence of psychiatric pathology and to the intervention of social, cultural, and emotional factors (Hill et al., 2020). In this regard, the results of recent research reveal that more than half of the cases with suicidal ideation and suicide attempts (62.36%) have a personal and/or inherited psychiatric pathological history (Ruderfer et al., 2020). Thus, attention should be drawn to the need to adequately study the history of each patient’s illness and the bioclinical particularities of the family environment in which they evolve (Sun & Zhang, 2018). Mental health experts and others must be extremely vigilant in identifying any reactions of patients that could “betray” the existence of suicidal ideation or a predisposition to self-harm (Benard et al., 2015; Ludwig & Dwivedi, 2018).

The accessibility of morbidity and mortality data also does not reflect the reality of the information present in the general population (Nigg, 2016; Forte et al., 2018). A first explanation could be that medical statistical systems often fail to capture those cases of suicide not effectively diagnosed by coroners (Lunde et al., 2018; Probert-Lindström et al., 2020). Another hypothesis is the use of poor-quality methods and means to quantify suicide attempts and suicidal ideation and/or self-harm (Rappaport et al., 2017).

Starting from the causal relationship between the interactions between cultural, social, family, and medical perspectives and the onset and maintenance of suicidal behaviour (Madsen et al., 2017; Bachmann, 2018), a detailed characterization of this interdependence can significantly improve the assessment of the main clinical-evolutional patterns (van Bergen et al., 2021).

Future research should focus on identifying and understanding the mechanisms underlying suicidal thoughts and behaviours specific to the adolescent population (MacArthur et al., 2018). These findings would positively and specifically influence specific dispositional changes such as impulsivity, aggressiveness, poor distress tolerance, and lack of social connectedness and family interactions (Ruderfer et al., 2020). These, in turn, may generate more effective treatment strategies in reducing suicidal thoughts and behaviours (van Bergen et al., 2021).

## 4. Conclusions

Overall, the issue of suicide is extremely complex and therefore there is likely to be a significant amount of under-reporting. However, suicides can be at least partially preventable by restricting access to the specific means of self-harm by training primary care physicians and health experts to identify people at risk and provide appropriate care, modulating the way it is reported in the media.

The presentation and characterization of the etiopathogenetic and clinical-evolutionary patterns of suicide facilitates the establishment of working hypotheses for future specialized research for identifying the best preventive strategies for this pathology.

Identifying and describing conditions conducive to an increase in the incidence and prevalence of psychological, social, intergenerational, or contextual factors associated with suicide allows for the much quicker establishment of specific prevention targets. At the same time, therapeutic interventions established by public health campaigns are also favoured.

## Figures and Tables

**Table 1:** Socio-cultural components of suicide (Durkheim theory).

Type of Suicide	Features
Selfish suicide	- Applicable to all those with poor integration in the social group to which they belong
	- May also explain the increased frequency of suicide among unmarried or poorly integrated families
Altruistic suicide	- Applicable to individuals who are susceptible to suicide, who have an excessive involvement in the group to which they belong
	- This is how suicide can be identified for the Japanese as a form of sacrifice in battle
Anomic suicide	- Applicable to individuals with a range of disorders that do not allow them to be truly integrated into a social group in terms of behaviour
	- Anomy describes a state characterized by constant social instability and a general disregard for social norms and values.

## Data Availability

Not applicable.
